# The Impact of Oral Sodium Chloride Supplementation on Thrive and the Intestinal Microbiome in Neonates With Small Bowel Ostomies: A Prospective Cohort Study

**DOI:** 10.3389/fimmu.2020.01421

**Published:** 2020-07-10

**Authors:** Tina Trautmann, Corinna Bang, Andre Franke, Deirdre Vincent, Konrad Reinshagen, Michael Boettcher

**Affiliations:** ^1^Department of Pediatric Surgery, University Medical Center Hamburg-Eppendorf (UKE), Hamburg, Germany; ^2^Institute of Clinical Molecular Biology (IKMB), Christian-Albrechts-University Kiel, Kiel, Germany

**Keywords:** small bowel ostomies, ileostoma, sodium chloride supplementation, failure to thrive, weight gain, microbiome, lactobacillus

## Abstract

**Background:** Infants with ileostomies often suffer from sodium depletion, ultimately leading to a failure to thrive. Moreover, early-infantile microbial dysbiosis may potentially aggravate weight faltering. Given that sodium supplementation has been used to restore weight gain and feeding practices largely determine infantile microbiota, the current study investigated the effect of sodium chloride (NaCl) on weight gain and intestinal microbiome in infants with jejuno- and ileostomies.

**Methods:** A prospective cohort study including 24 neonates with enterostomies compared 19 subjects receiving oral NaCl (5.85%) to five subjects without supplementation with respect to postoperative changes in thrive and the intestinal microbiome.

**Results:** Infants receiving NaCl after enterostomy-surgery showed vastly improved weight gain and an increased abundance of *Lactobacillus* in fecal samples, as compared to subjects without oral supplement who displayed decreasing percentiles for weight and did not reveal a higher abundance of probiotic strains within the ostomy effluent. Contrarily*, Klebsiella* was equally enriched in supplemented infants, reflecting a higher susceptibility for infections in preterm neonates.

**Discussion:** Our findings support oral NaCl supplementation as a mainstay of postoperative treatment in infants with small bowel ostomies who are predisposed to suffer from a sodium depletion-associated failure to thrive. Not only does NaCl promote weight gain by increasing glucose resorption, but it also appears to induce microbial restoration by enhancing the abundance of health-promoting probiotic bacteria. This finding has an even greater significance when facing an elevated *Klebsiella/Bifidobacteria* (K/B) ratio, believed to represent an early-life microbial biomarker for development of allergic disease.

## Background

Temporary enterostomies following bowel resection are a common procedure in the surgical treatment of severe gastrointestinal diseases, such as the necrotizing enterocolitis (NEC)—one of the most common and devastating neonatal surgical emergencies (1–3). Thus, temporary enterostomies are often performed on premature and newborn infants, which are predisposed to severe fluid losses and electrolyte disturbances (1, 4). Consequently, these infants often suffer from a total body sodium depletion (Na^+^ <10 mEq/L in spot urine) and severe hydrogencarbonate losses (HCO^3−^ <20 mEq/L in serum), ultimately leading to a failure to thrive (weight <5th percentile) and a metabolic acidosis (5–8). Weight faltering following stoma formation poses a devastating enterostomy-associated complication, correlated with a higher CDC-grade, need for reoperation, and early stoma reversal (4). Oral sodium supplementation has been reported to support these infants in regaining weight (5, 6). Previous studies explained the failure to thrive by an ineffective intestinal sodium-glucose cotransport (SGLT-1) or an overall shut-down of anabolic pathways (5–7, 9). Yet, the most reasonable explanation seems to be a mixture of various deregulated mechanisms generated by the observed sodium depletion. Hence, sodium supplementation has been suggested as the best therapeutic means to restore appropriate weight gain in infants with small bowel ostomies (5–7).

The human body's microbiota essentially contributes to its immune status, central nervous system, bowel development, intestinal digestion, and general metabolism (10–14). Thus, underweight infants in particular may benefit from the microbial's ability to extract energy from otherwise indigestible dietary polysaccharides (15). More specifically, a newborn's microbiome resembles their maternal stool, vaginal, and/or skin microbiota and continues to be colonized by parental oral and skin microbes (16–18). Facultative anaerobes typically first colonize the infant's intestine, followed by obligate anaerobes during the subsequent 6 months of life (19). The microbiota's diversity remains narrow in early infancy and is dominated by species involved in human milk oligosaccharide (HMO) metabolism of breastfed infants (20). In fact, the small intestine represents a unique environment as food retention is relatively short and microbial growth is largely hampered. Meanwhile, the abundance of simple carbohydrate transport phosphotransferase systems (PTS) indicates that rapid uptake and fermentation of available carbohydrates contributes to the microbiota maintenance (12, 21).

To date, no research has been published about the impact of sodium supplementation on the microbiome of the small intestine. Supposing sodium activates intestinal SGLT-1, which in turn leads to an increased glucose resorption, we hypothesize that less simple carbohydrates are available to be fermented by the present microbes, thus, causing the microbiota to adapt by extending their presence in this part of the intestine. Being particularly linked to weight gain, the microbiome could counteract weight faltering in predisposed infants (22). Thus, the main purpose of the current study was to evaluate the effects of sodium supplementation on weight gain and the intestinal microbiome in infants with small bowel ostomies.

## Results

A total of 24 patients were included in the current study and divided into two groups (NaCl vs. control) of whom 19 infants (19/24) received 5.85% sodium chloride (5.8 (3.0–10.4) ml) and five neonates (5/24) were not orally supplemented. Patient characteristics relevant with respect to the infants' preoperative bowel condition and anticipated postoperative complications are summarized in Table 1. Birth weight (*p* = 0.095), gestational age (*p* = 0.057), and bowel length until stoma (*p* = 0.18) did not differ significantly between groups.

**Table 1:** Summary of demographic and clinical patient characteristics.

**Patient data**	**NaCl *(N)***	**Control *(N)***
Supplementation	19	5
Gender		
Female	8 (42%)	1 (20%)
Male	11 (58%)	4 (80%)
Gestation week	26 (24–32)	32 (31–40)
Preterm infants	15 (79%)	3 (60%)
Birth weight (g)	820 (660–2,760)	1,820 (1,080–3,425)
Percentiles	53 (31–79)	54 (22–72)
Diagnosis		
Necrotizing enterocolitis	8 (42%)	1 (20%)
Meconium ileus	4 (21%)	3 (60%)
Hirschsprungs disease	4 (21%)	1 (20%)
Volvulus	2 (11%)	
Anal atresia	1 (5%)	
Osteotomy		
Bowel length to ostomy (cm)	73 (55–129)	129 (73–196)
Bowel resection	4 (21%)	1 (20%)
Jejunostoma	2 (11%)	1 (20%)
Ileostoma	17 (89%)	4 (80%)
5.85% NaCl Amount (ml)		
Median (IQR)	5.8 (3–10.4)	

### Weight Gain

**Figure 1:**
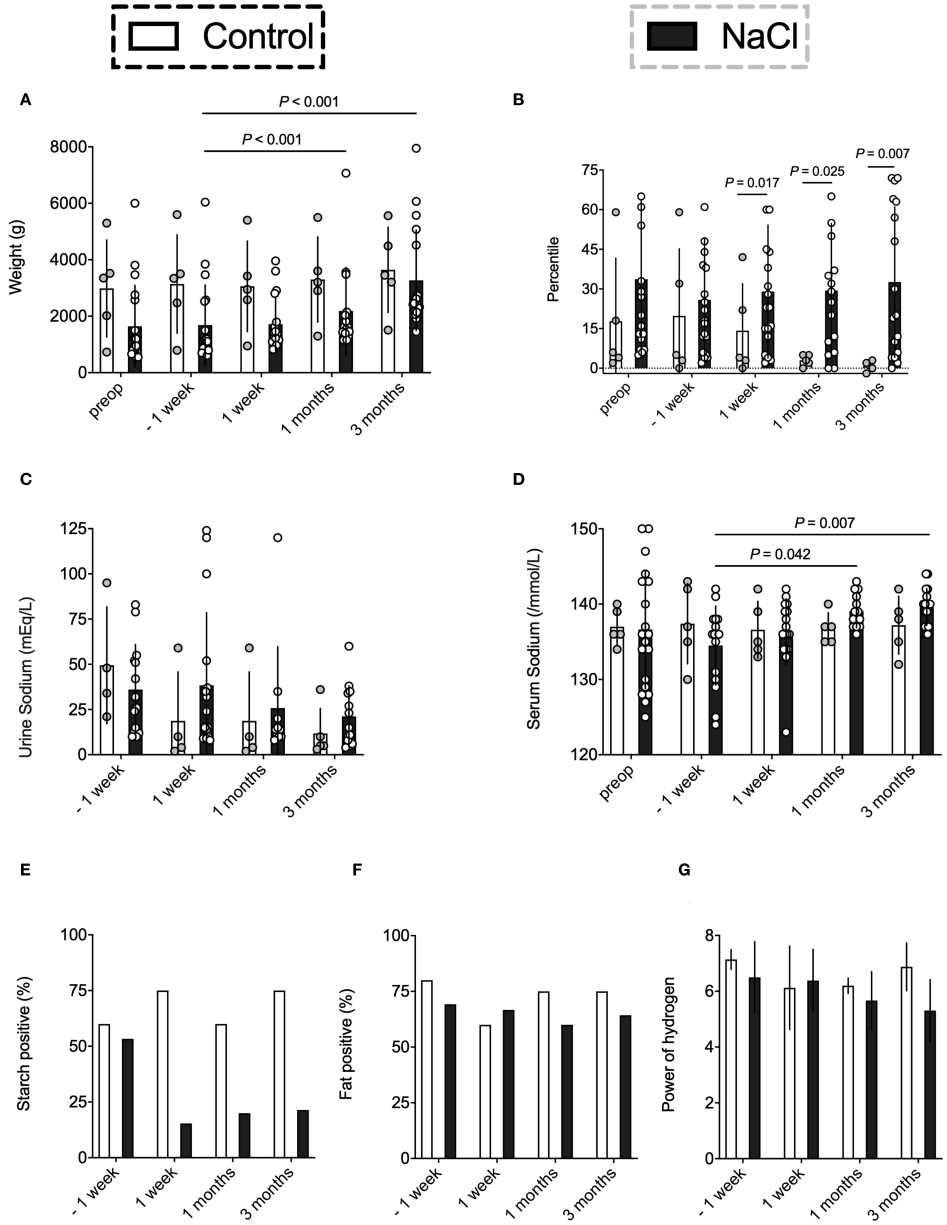
Evaluation of the effect of NaCl on weight gain, sodium levels, and resorptive bowel capacity. Data were analyzed on five occasions: before stoma formation (preop), 1 week before intervention* (−1 week), and on three occasions after intervention* (1 week, 1 month, 3 months). *intervention = NaCl vs. none. **(A,B)** The NaCl group revealed an improved weight gain after supplementation as compared to the control group who showed steadily decreasing Fenton percentiles. **(C,D)** Urine sodium levels were quite similar in both groups. However, the NaCl group revealed higher serum sodium levels at one and three months after stoma formation. **(E)** The NaCl group showed an improved starch resorption after supplementation as compared to the control group. A reduction in starch losses reflects an increased glucose resorption due to an activation of intestinal SGLT-1. **(F)** Fatty acid positive effluent was not affected by supplementation. **(G)** The NaCl group showed mean decreasing pH levels over time as compared to the control group. Alkaline pH might indicate a shift to acid producing bacteria like *E. coli* (23) and/or arise from profound changes in microbiota assembly (e.g., increase of *Lactobacillaceae*) (24).

The effect of NaCl on weight gain was analyzed in both groups (Figures 1A,B). All infants had a comparable base line weight and showed a steady weight increase throughout the 3 months after stoma formation. However, the NaCl group demonstrated a much better improvement as compared to the control group (Figure 1A). Moreover, at the beginning of the study, subjects of both groups presented similar percentile ranges with respect to weight. Over the course of the study, however, the NaCl group showed an improvement over time, whereas the percentiles in the control group decreased steadily (Figure 1B).

### Sodium Levels

Mean sodium levels were analyzed in spot urine, which most accurately describe the actual sodium intake (5, 6). As expected, the NaCl group showed higher urine sodium levels, yet, they did not differ significantly as compared to the control group. However, infants without supplementation exhibited a constant decrease of urine sodium level output after stoma formation (Figure 1C). More precisely, 4/5 infants in the control group showed urine sodium levels below 10 mEq/L, whereas the NaCl group had only 5/14 infants with inadequate sodium intake.

Sodium supplementation did affect serum sodium levels in the NaCl group, with significant increases in sodium levels at 1 and 3 months after stoma construction (Figure 1D). Meanwhile, the control group had steady serum sodium levels of 136–137 mEq /L throughout the entire postoperative phase. No significant differences between groups were observed.

### Resorptive Capacity

Postoperative resorptive bowel capacity in both groups was measured by evaluating the ostomy effluent in regard to changes in starch, fatty acids, and pH (Figures 1E–G). Carbohydrates are mainly absorbed in the mid jejunum, which was preserved in most subjects after surgery. Thus, starch positive effluent could be used as a rough indicator for increased starch losses and might correlate with decreased bowel resorption capacity (26). The NaCl group showed an improved starch resorption after supplementation, whereas the control group exhibited high starch losses in more than half of the subjects (Figure 1E). Contrarily, fatty acid positive effluent was not affected by sodium intake (Figure 1F). Moreover, the NaCl group showed mean decreasing pH levels over time as compared to the control group, possibly indicating a shift to acid producing bacteria like *E. coli* or *Klebsiella* (23) (Figure 1G).

### Effect of Time on ß-Diversity

**Figure 2:**
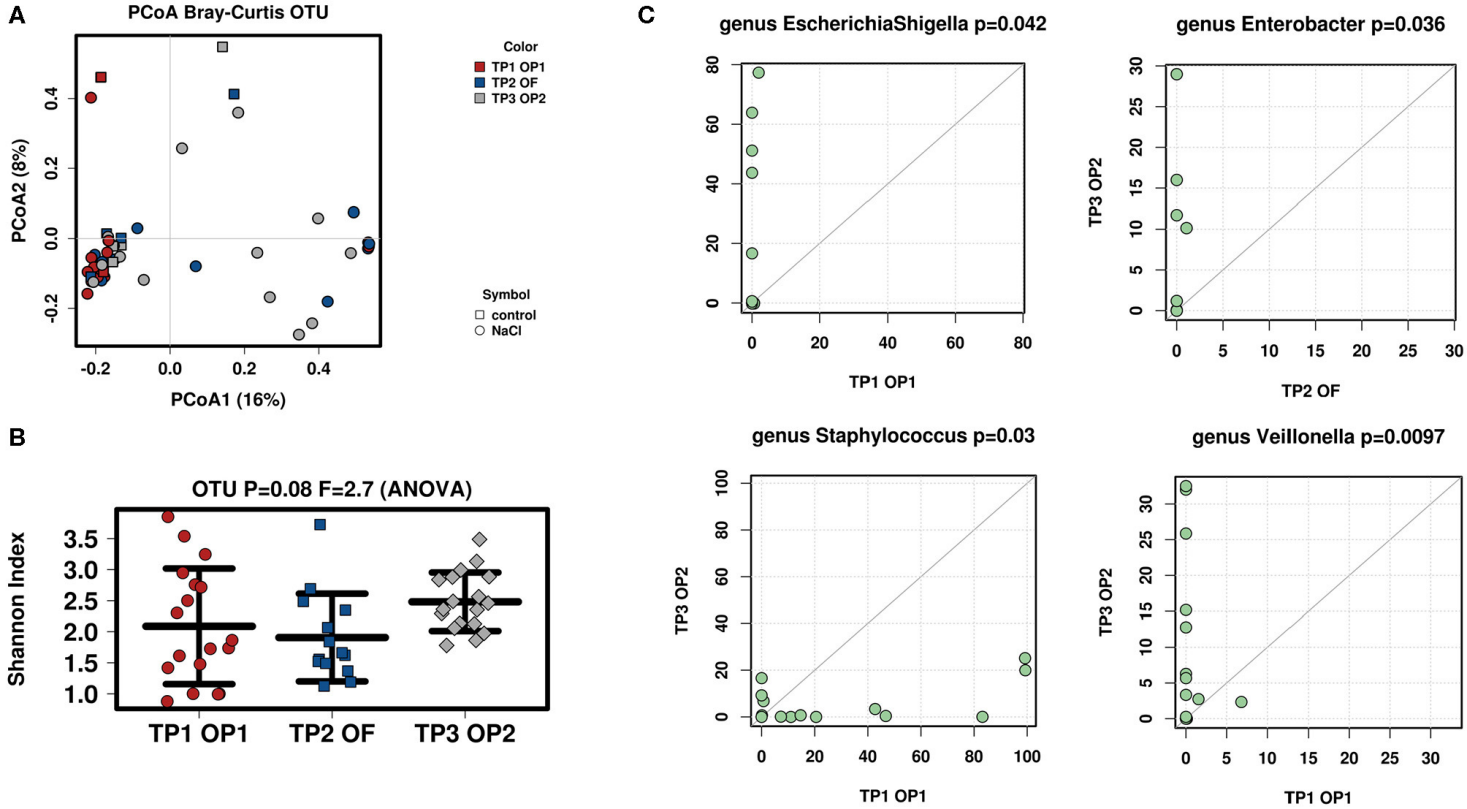
Analysis of microbial diversity and taxa abundance as to changes in time (TP1 OP1, TP2 OF, TP3 OP2). **(A)** PCoA of all samples was performed to evaluate ß-diversity of observed OTUs: Microbiota composition between all infants became increasingly diverse with time (TP1 OP1–TP3 OP2). **(B)** Shannon-Index was used to analyze α-diversity at OTU-level (read depth was normalized to 1321): Microbiota composition within infants showed a fluctuating diversity at different points in time (TP1 OP1, TP2 OF, TP3 OP2). Increasing interindividual diversity and fluctuating intraindividual variation may arise from individually distinct influences on the recovering intestine. **(C)** Total-sum scaling (TSS) was applied to evaluate taxa abundance at genus-level. Significant differences were seen in *EscherichiaShigella* (TP1 OP1 vs. TP2 OF), *Staphylococcus* (TP1 OP1 vs. TP3 OP2), *Enterobacter* (TP2 OF vs. TP3 OP2), and Veillonella (TP1 OP1 vs. TP3 OP2). The successive increase of *Enterobacteriaceae* may be explained by ongoing contact of the stoma to oxygen (25). A simultaneous decrease in *Staphylococcus* might be provoked by postoperative recovery of the surgery wound (25).

Interindividual variation (ß-diversity) in microbiota composition within the ostomy effluent of all infants was assessed with respect to point in time (TP1 OP1, TP2 OF, TP3 OP2) using a Bray-Curtis Dissimilarity Measure for analysis of operational taxonomic units (OTUs) (Figure 2A). Data sets were examined for clusters using a Principle Component Analysis (PCoA). All infants showed a similar microbial composition after stoma formation (TP1 OP1). However, microbial between-sample variation increased significantly after oral feeding (TP2 OF) and before stoma reversal (TP3 OP2).

### Effect of Time on α-Diversity

Intraindividual variation (α-diversity) in microbiota composition of all infants' ostomy effluent was again evaluated regarding point in time (TP1 OP1, TP2 OF, TP3 OP2) using the Shannon-Index for analysis of OTUs (Figure 2B). Results revealed a clear trend toward a high diversity of microbial species when examining the above-mentioned consecutive samples (*p* = 0.08). Species diversity was highest after stoma formation (TP1 OP 1) and before stoma reversal (TP3 OP 2). Meanwhile, microbial species were found to be distributed more evenly in all samples after initiation of oral feeding (TP2 OF).

### Effect of Time on Microbial Abundance

The Wilcoxon-rank test was then used to the evaluate microbial abundance of all infants' fecal samples regarding point in time (TP1 OP1, TP2 OF, TP3 OP2; Figure 2C). Stool samples were compared as to whether their bacterial abundance mean ranks differed between two respective points in time (TP1 OP1 vs. TP2 OF, TP1 OP1 vs. TP3 OP3, TP2 OF vs. TP3 OP3). Significantly different taxa at genus-level are shown as a TaxaScatter. A total of 115 genera were identified in analyzed samples. The most abundant genera included *Staphylococcus* and *Lactobacillus* after stoma formation (TP1 OP1), *Staphylococcus, Escherichia/Shigella, Lactobacillus, Enterococcus, Streptococcus* and *Klebsiella* after initiation of oral feeding (TP2 OF), and *Veillonella, Enterococcus, Streptococcus* and *Klebsiella* before stoma reversal (TP3 OP2). The most apparent finding was a postoperative inverse dynamic of *Staphylococcus* vs. *Escherichia/Shigella*. More specifically, *Escherichia/Shigella* significantly increased (*p* = 0.042) while *Staphylococcus* significantly decreased (*p* = 0.03) from stoma formation (TP1 OP1) toward oral feeding (TP2 OF) and stoma reversal (TP3 OP2). Moreover, *Enterobacter* significantly increased from oral feeding (TP2 OF) until stoma reversal (TP3 OP2) (*p* = 0.036) and *Veillonella* significantly increased from stoma formation (TP1 OP1) until stoma reversal (TP3 OP2) (*p* = 0.0097).

### Effect of Treatment on ß-Diversity

**Figure 3:**
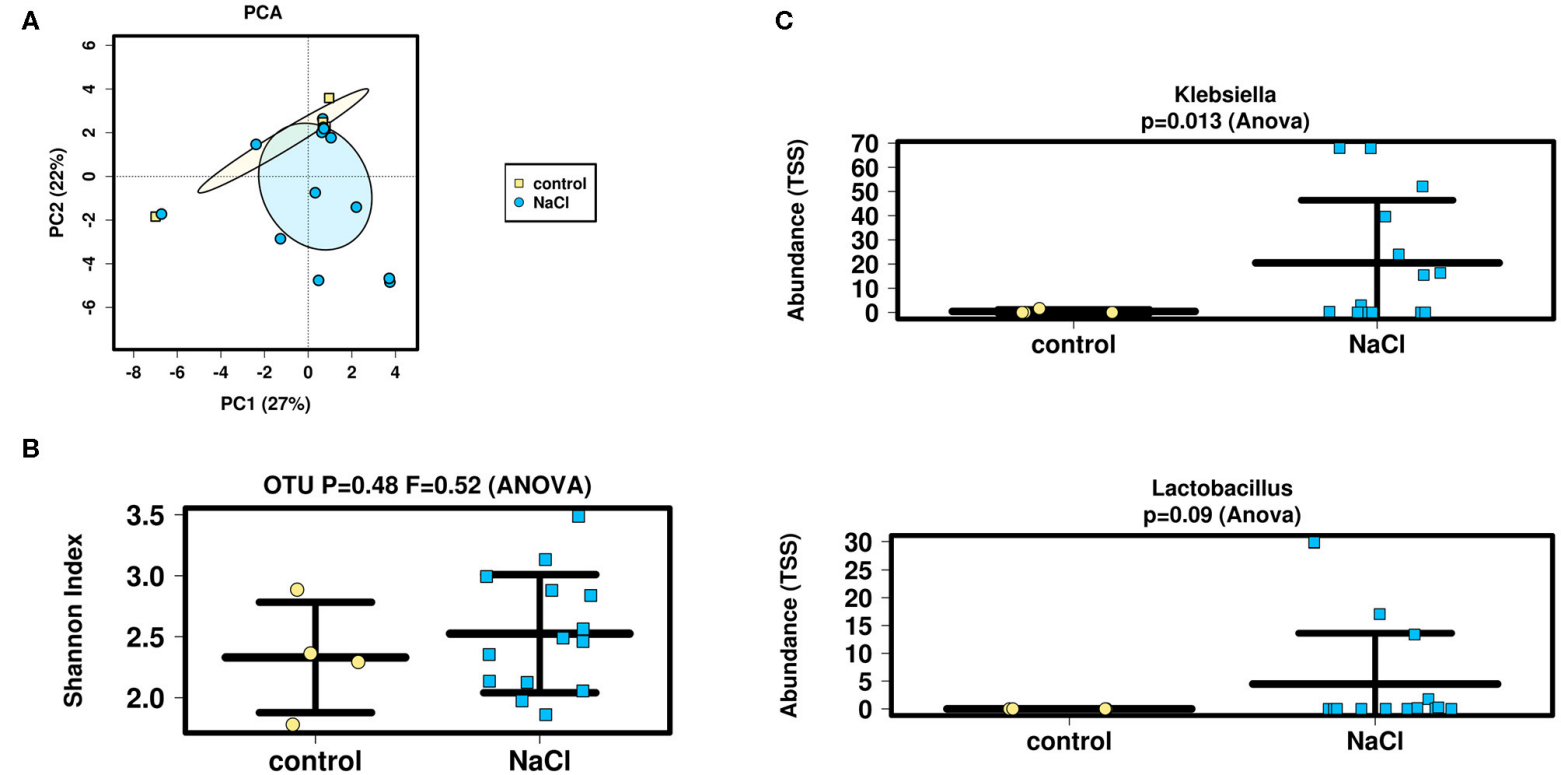
Analysis of microbial diversity and taxa abundance as to treatment (NaCl vs. control) **(A,B)** PCA and Shannon-Index were performed/applied on/to all samples to evaluate ß- and α-diversity at OTU-level (sequencing depth normalized to 1,911 reads): No significant differences were detected as to inter- and intraindividual microbial variation between groups. **(C)** TSS was applied to evaluate taxa abundance at genus-level. Analysis showed an increased abundance of *Lactobacillus* and significantly higher abundance of *Klebsiella* in the NaCl group. The increase in *Lactobacillus* strains might indicate a health-promoting effect of NaCl on the intestinal microbiome. Higher abundance of *Klebsiella* is suggested to arise from an increased susceptibility for infections of predominantly premature infants within the NaCl group.

Interindividual variation (ß-diversity) in microbiota composition of infants' samples was then assessed as to differences between groups (NaCl vs. control) before stoma reversal (TP3 OP 2) using a Bray-Curtis Dissimilarity Measure for analysis at OTU-level (Figures 3A). Data sets were examined for clusters using a Principle Coordinate Analysis (PCA) and results revealed microbial between-sample variation was not altered significantly in either of the groups.

### Effect of Treatment on α-Diversity.

Intraindividual variation (α-diversity) in microbiota composition of these samples was then evaluated using the Shannon-Index at OTU-level (Figure 3B). Again, no significant difference between groups (NaCl vs. control) could be detected. Moreover, the genera-count revealed similar numbers of detectable genera (mean = 16).

### Effect of Treatment on Microbial Abundance

Finally, ANOVA analysis was performed at genus level to compare fecal samples with respect to bacterial abundance regarding treatment (NaCl vs. control) before stoma reversal (TP3 OP 2) (Figure 3C). Taxa data were compared by nested analysis of variance. A total of 115 different genera from 70 families were identified in the analyzed samples and the most abundant families in fecal samples included *Lactobacillaceae* and *Enterobacteriaceae*. Analyses revealed a slightly higher abundance of *Lactobacillaceae* in the NaCl group (*p* = 0.35), whereas no difference was detected between groups regarding *Enterobacteriaceae* (*p* = 0.27).

The most abundant genera in effluent samples were *Klebsiella, Veillonella, Raoultella*, and *Escherichia/Shigella*. Analyses showed a significantly higher abundance of *Klebsiella* (*p* = 0.013) and a slightly higher abundance of *Lactobacillus* in the NaCl group (*p* = 0.09). Meanwhile, no difference could be detected between groups regarding *Escherichia/Shigella* (*p* = 0.72), *Raoultella* (*p* = 0.79), and *Veillonella* (*p* = 0.45).

## Discussion

Previous studies have shown that neonates with small bowel ostomies often suffer from a total body sodium depletion ultimately leading to a failure to thrive (5–8). Studies based on clinical experience revealed that sodium administration improves electrolyte disturbances and promotes weight gain in affected infants. Thus, the current study was performed to scientifically analyze the impact of sodium supplementation on weight gain in infants with jejuno- and ileostomies. Our results suggest that neonates who received 5.85% oral NaCl supplementation after surgery showed a vastly improved weight gain as compared to the control group. Meanwhile, their serum sodium levels increased and remained stable within the physiological range. In contrast, newborns without NaCl supplementation mapped on much lower weight percentiles and eventually were categorized as a failure to thrive. Moreover, a constant decrease of urine sodium levels was noticed in both groups, with bigger sodium losses observed in the control group. Microscopic analyses and pH measurements of the ostomy effluent showed significantly lower starch losses and pH levels in supplemented infants as compared to the control group. The observed differences amongst both groups can most likely be explained by a sodium-dependent activation of intestinal SGLT-1, leading to an increase in duodenal glucose absorption capacity, which not only corrects electrolyte imbalances, but also induces weight gain. In fact, the perceived decreases in starch losses within the ostomy effluent likely reflected an increased glucose resorption in the NaCl group. Additionally, the alkaline pH might indicate a shift to acid producing bacteria like *E. coli* (23) and/or arises from profound changes in microbiota assembly (e.g., increase of *Lactobacillaceae*) (24). Infants with a short bowel syndrome postoperatively often depend on total parental nutrition (TPN), which in turn is associated with an increased risk for sepsis, cholestasis, and liver failure (27). However, the bowel remnant is believed to have some potential to functionally adapt in response to absorptive surface losses after extensive resection (28, 29). Hence, further studies could investigate whether sodium enhanced postoperative bowel adaptation, since an early transition to an oral diet could reduce TPN associated complications.

An “undernourished” microbiome and disruptive succession in early infancy has been associated with severe and lifelong growth deficits and neurodevelopmental impairment (17). Especially dysbiosis of the small intestinal microbiome, which is known to essentially account for nutrient absorption, is believed to contribute to impaired development (17). Previous studies investigating the ileal physiological and healthy microbiome of adults, revealed a reduced diversity and high temporal fluctuations in the microbial community. Moreover, most abundant genera were found to be *Streptococcus* and *Veillonella* (30). Since research on the small intestinal microbiota composition of infants is scarce, our study also analyzed the microbial diversity and taxa abundance of neonatal patients with respect to time (TP1 OP1, TP2 OF, TP3 OP2), Analyses revealed that interindividual variation (ß-diversity) in microbiota composition between all infants became increasingly diverse from stoma formation (TP1 OP1) until stoma reversal (TP3 OP2). Moreover, intraindividual variation (α-diversity) within each infant's microbial community had a temporarily fluctuating diversity at different points in time (TP1 OP1, TP2 OF, TP3 OP2). The most abundant small intestinal microbes at genus level were *Staphylococcus* and *Lactobacillus* (TP1 OP1), *Staphylococcus, Escherichia/Shigella, Lactobacillus, Enterococcus, Streptococcus* and *Klebsiella* (TP2 OF), and *Veillonella, Enterococcus, Streptococcus* and *Klebsiella* (TP3 OP2). The increasing diversity and temporal fluctuations in microbial community may be explained by individually distinct influences on the recovering intestine (e.g., healing process of operated bowel, resorptive adaptation of remaining intestine, feeding practices, and antibiotic exposure). The procession of early colonization with facultative anaerobes (e.g., *Staphylococcus)* followed by an abundance of obligate anaerobes (e.g., *Veillonella*) resembles the microbial assembly in colonic samples of healthy neonates (19). Additionally, most species identified were related to cesarean delivery (i.e., *Staphylococcus, Streptococcus, Veillonella*), while less species were associated with vaginal delivery (i.e., *Enterobacteriaceae*) (18). The successive decrease in *Staphylococcaceae*, predominantly found on skin and mucosa, might be provoked by the postoperative recovery of the surgery wound (25). Moreover, the concurrent increase of facultative anaerobe *Enterobacteriaceae* may be explained by the ongoing contact of the stoma to oxygen (25). Finally, higher levels of *Staphylococcaceae* and low abundance of *Bifidobacteria* are associated with the predominance of premature infants in the current study (31). Further insights on how microbes cooperate or compete in a changing intestinal environment would be useful to distinguish beneficial from unfavorable influences.

Malnutrition in early infancy essentially contributes to microbial dysbiosis, provoking a disruption of bowel integrity, digestion, absorption, and energy storage (17). Especially preterm infants with small bowel ostomies, who often suffer from a failure to thrive, may be affected by complications of dysbiosis. Various studies have identified the type of feeding to highly influence the colonic microbiome in infants (15, 17–19). For instance, breast-feeding appears to have a favorable impact on the early infant microbial assembly. In fact, the most abundant bacteria in breast-fed infants of maternal secretors are *Bifidobacteria*, which are known to be beneficial for intestinal health (17). However, research investigating the effect of the type of feeding on the small intestinal microbiome is scarce. As the beneficial properties of sodium might arise from its effect on the small intestine, microbial diversity and taxa abundance were compared between groups (NaCl *vs*. control). Analysis of ß- and α-diversity revealed no significant difference as to inter- and intraindividual species variation between groups. However, analysis on taxa abundance showed an increasing abundance of *Lactobacillus* and a significantly higher abundance of *Klebsiella* in the NaCl group as compared to the control group. Thus, sodium supplementation might have a favorable impact on the intestinal microbiome by increasing the abundance of health-promoting probiotic strains such as *Lactobacillaceae*, though this finding has to be validated in a follow-up study including a larger number of individuals and more equally distributed groups. Supposing that sodium increases glucose resorption, less carbohydrates are available to be fermented by microbes. Hence, microbial enrichment of the intestine may be explained by a reactive adaptation process to low carbohydrate levels. Contrarily, *Klebsiella* known to be an opportunistic pathogen, was equally enriched in supplemented infants. One explanation for this finding might arise from the fact that supplemented neonates in this study were mostly premature infants with very low birth weight (VLBW). As these patients frequently suffer from a compromised immune system, increased levels of opportunistic pathogens most likely emerge from higher susceptibility for infections. Recent research on the abundance of *Klebsiella* in infantile microbiota suggests that an elevated *Klebsiella/Bifidobacteria* (K/B) ratio might represent an early-life microbial biomarker for development of allergic disease (32). Hence, concurrent enrichment of the infantile microbiota with probiotic strains (i.e., Bifidobacteria) has an even greater significance, as an abundance of *Klebsiella* could predispose individuals for development of childhood allergies.

Based on our results, we highly recommend monitoring of sodium losses in all children with enterostomies to include measurement of urine sodium levels, as solely tracing serum sodium levels appears to be insufficient when aiming to detect an arising sodium depletion. Infants without NaCl supplementation showed steadily decreasing urine sodium levels and eventually suffered from weight faltering. Persistent low urine sodium levels below 10 mEq/L account for an inadequate sodium intake and should be supplemented immediately to prevent a failure to thrive. Children with ileostomies have a basal sodium need of 1.2 mEq/kg/day. In most subjects 4–6 mEq/kg/day NaCl will be sufficient. However, sodium levels should be monitored via spot urine to remain above 10 mEq/L (5).

Limitations of the current study include (1) the small number of subjects, (2) patient differences between groups, (3) external influences affecting the microbiota, and (4) the low content of fecal samples. Infants included in this study suffered from a small subset of severe intestinal diseases, which mostly affect extremely premature infants with substantial comorbidities. Hence, recruiting a sufficient number of subjects to strengthen the overall results has generally been challenging. Moreover, groups differed as to gestational age, birth weight, bowel length to ostomy, and overall health status. A larger sample size could have further reduced observed differences to improve comparability between groups. Additionally, early microbiota is influenced by numerous external factors (e.g., birth route, hygiene, stress, nutrition, drugs) (33). This study specifically examined the influence of sodium on the microbiome. However, concurrent factors such as the broad use of antibiotics need to be taken into account. Likewise, renal failure represents a common complication in children with ileostomies, hence, a deteriorated kidney function should be considered to affect urine sodium levels. Due to the low number of DNA in the neonatal effluent, a cut-off filtering of 1,000 reads was used for 16s rRNA sequencing. To reduce the risk for associated sequencing background, controls of DNA preparation (PrepCo), as well as negative controls, were run during sequencing.

Collectively, our findings support oral sodium supplementation as a mainstay of postoperative treatment in infants suffering from a failure to thrive after stoma formation. Not only does sodium application promote weight gain in those infants, but it also appears to have a favorable impact on maintaining the microbiota within the infant's small intestine.

## Materials and Methods

### Study Design

The study was designed as a prospective study involving the Children's Hospital Altona and the Department of Pediatric Surgery of the University Medical Center Hamburg-Eppendorf. All neonates with jejuno- or ileostomy creation between August 2017 and August 2019 were included. The study was conducted in accordance with guidelines of the medical research ethics committee of Hamburg (Ethik-Kommission der Ärztekammer Hamburg, PV5673) as well as the 1964 Helsinki declaration and its later amendments. Informed consent was obtained from parents or legal guardians.

### Subject Data

All patient information was prospectively recorded including gender, gestational week, birth weight, reason for stoma formation, bowel length until stoma, whether bowel was resected, type of stoma, and amount of NaCl supplement (Supplement 1).

### Sodium Supplementation

A total body sodium depletion is defined as Na^+^ <10 mEq/L in spot urine, yet, serum sodium levels may still lay within the physiological range (135–145 mEq/L) (5, 6). At our clinic, sodium levels are routinely monitored in blood serum or arterial blood gas (ABG), hence, we additionally measured urine sodium levels in observed neonates. Infants received a calculated TPN, which covers the basal sodium need of 2–4 mEq/kg/day. Additional oral sodium was administered to infants who presented with hyponatremia (Na < 135 mEq/L) on repeated blood draws (serum or ABG). Neonates initially received 1 mEq/kg/dose 5.85% NaCl orally 2–4 times a day. The dosage was then adjusted aiming for sodium levels in spot urine to remain above 10 mEq/L. The decision on whether infants were supplemented or not, was based on the preference of the responsible attending physician and not on blood or urine sodium levels.

### Data Collection

Routine urine and blood sampling data was recorded to determine each participant's sodium levels. Electrolyte levels were measured according to standardized laboratory methods at our hospital (potentiometric method). Additionally, overall weight and percentile trends were monitored to evaluate changes in thrive. Weight was recorded by the nurse staff of our hospital and percentiles were calculated according to the Fenton Growth Chart (34). Finally, two 1 mL aliquots of fecal samples were withdrawn from the ostomy effluent. One aliquot was used for analysis of postoperative bowel resorption capacity, while the other aliquot was examined to characterize the microbiome at the stoma site. Since the main objective of this study was to assess the impact of sodium supplement on weight gain and microbial changes, data/samples of both groups (NaCl vs. control) were derived/withdrawn before and after intervention (sodium vs. none) between stoma formation and ostomy reversal (TP1 OP1 TP3 OP2). An overview of data collection is provided in an according timeline (Supplement 1).

### Stool Sampling

Most infants were monitored and treated at the neonatal or pediatric intensive care unit after surgery. Here, only medical staff and legal guardians were allowed access to the patients. This ensured adequate hygienic and aseptic handling when taking care of the infants. Daily stoma care included the aspiration of fecal effluent every 6–8 h and the change of the ostomy bag after 3 days at the latest. Infants either received ileal or jejunal double-barrel ostomies. When two enterostomies were constructed, samples were derived from the higher/orally located stoma. Fecal effluent was collected from the ostomy bag to a coded tube using a sterile syringe. Within 2 h, fecal tubes were frozen down to −80°C at the laboratory. After all samples have been collected, they were placed on dry ice for further analyses. One aliquot was used to evaluate semi-quantitative changes in intestinal nutrient resorption capacity at an external laboratory. Here, fecal samples were analyzed microscopically for starch positive effluent (Lugol's iodine dye) and fatty acid losses (Sudan stain). Fecal pH was measured using a regular pH indicator strip (MERCK pH-Universal-Indicator 1-10). The other aliquot was used for microbiota analysis (16S rRNA sequencing) at IKMB of the University of Kiel.

### 16S rRNA Sequencing

DNA was extracted from fecal samples using the QIAamp DNA fast stool mini kit automated on the QIAcube (Qiagen, Hilden, Germany). Therefore, material was transferred to 0.70 mm Garnet Bead tubes (Dianova, Hamburg, Germany) filled with 1.1 mL InhibitEx lysis buffer. Bead beating was performed using the a SpeedMill PLUS (Analytik Jena, Jena, Germany) for 45 s at 50 Hz. Samples were then heated to 95°C for 5 min with subsequent continuation of the manufacturer's protocol. Extracted DNA was stored at −20°C prior to PCR amplification. Blank extraction controls were included during extraction of samples.

For sequencing, variable regions V1 and V2 of the 16S rRNA gene within the DNA samples were amplified using the primer pair 27F-338R in a dual-barcoding approach according to Caporaso et al. (35). Three microliter stool DNA were used for amplification. PCR-products were verified using the electrophoresis in agarose gel. PCR products were normalized using the SequalPrep Normalization Plate Kit (Thermo Fischer Scientific, Waltham, MA, USA), pooled equimolarily and sequenced on the Illumina MiSeq v3 2x300bp (Illumina Inc., San Diego, CA, USA). Demultiplexing after sequencing was based on 0 mismatches in the barcode sequences.

### Statistical Analysis

Clinical data was analyzed using SPSS Statistics 26 (IBM, NY, USA) and GraphPad Prism 8 (GraphPad, CA, USA). The power was concluded from a previous study examining the influence of NaCl on weight gain in children (5). Differences between groups were calculated using the Mann-Whitney test. For nominal data, Fisher's exact test was used. To evaluate repeated measures mixed effect model with Dunnett correction were used.

Microbiome data processing was performed using the DADA2 workflow for big datasets resulting in abundance tables of amplicon sequence variants (ASVs) (36). All sequencing runs were handled separately and were collected in a single abundance table per dataset, which underwent chimera filtering. ASVs underwent taxonomic annotation using the Bayesian classifier provided in DADA2 and the Ribosomal Database Project (RDP) version 16 release. ASV abundance tables and taxonomic annotation were passed on to the phyloseq package for construction of phylum- to genus-level abundance tables. Sequences that were not assignable to genus level were binned into the finest-possible taxonomic classification. The web-based software Calypso version 7.14 was used for analyses of microbiome data from samples with a cut-off of 1.000 clean reads (37). A brief overview of sequencing reads obtained for analyzed samples is provided in Supplementary Figure 1. Microbiome alpha diversity measures were estimated using Shannon index. These measures were calculated on rarefied ASV table sequences per sample (1,321 for all samples in Figure 3B, 1,911 for samples of TP3 OP2 in Figure 2A). *P*-values were calculated using ANOVA. Beta diversity was calculated on rarefied ASV table with Bray-Curtis dissimilarity. Principal coordinate analyses were applied to visualize the level of dissimilarity of samples (Figure 3A). An overview of the microbial community composition of the most abundant bacterial families is provided for all samples (Supplementary Figure 2). LDA Effect Size (LEfSE) was applied to identify differently abundant taxa at different points in time (Supplementary Figure 3). Significant differences between points in time were calculated using the Wilcoxon-rank test at genus level. The level of significance was set at 0.05.

## Data Availability Statement

All datasets generated for this study are included in the article/Supplementary Material.

## Ethics Statement

The studies involving human participants were reviewed and approved by Ethik-Kommission der Ärztekammer Hamburg, PV5673. Written informed consent to participate in this study was provided by the participants' legal guardian/next of kin.

## Author Contributions

TT conceptualized and designed the study, acquired and analyzed clinical data, drafted the initial manuscript, and approved the final manuscript as submitted. CB and AF acquired and analyzed microbiome data and approved the final manuscript as submitted. DV acquired and analyzed clinical data, drafted the initial manuscript, and approved the final manuscript as submitted. KR conceptualized and designed the study and approved the final manuscript as submitted. MB conceptualized and designed the study, acquired and analyzed clinical data, performed statistics, drafted the initial manuscript, and approved the final manuscript as submitted. All authors contributed to the article and approved the submitted version.

## Conflict of Interest

The authors declare that the research was conducted in the absence of any commercial or financial relationships that could be construed as a potential conflict of interest.

## Supplementary Material

Supplement 1 Timeline of sample and data collection. Red: Fecal samples (resorption, microbiota) were withdrawn on three occasions: after stoma formation (TP1 OP 1), after initiation of oral feeding (TP2 OF), and before stoma reversal (TP3 OP2). Blue: Growth related data (sodium, weight) were documented on five occasions: before stoma formation (preop), 1 week before intervention^*^ (−1 week), and on three occasions after intervention^*^ (1 week, 1 month, 3 months). ^*^intervention = NaCl vs. none.

Supplementary Figure 1 Number of sequence reads per sample. Brief overview of sequencing reads obtained for samples analyzed in this study.

Supplementary Figure 2 Quantitative visualization of community composition at family level. Microbial community composition including the 100 most abundant bacterial families is shown for individual samples.

Supplementary Figure 3 Feature selection for points of time. Bacterial OTUs associated with different points of time were identified by using the linear discriminant analysis (LDA) effect size method (LEfSe) implemented in Calypso Web version.

## References

[1] Steinau G Ruhl KM Hornchen H Schumpelick V. Enterostomy complications in infancy and childhood. Langenbecks Arch Surg. (2001) 386:346–9. 10.1007/s004230100243 11685565

[2] Neu J. Neonatal necrotizing enterocolitis: an update. Acta Paediatr Suppl. (2005) 94:100–5. 10.1111/j.1651-2227.2005.tb02163.x 16214774

[3] Pierro A Hall N. Surgical treatments of infants with necrotizing enterocolitis. Semin Neonatol. (2003) 8:223–32. 10.1016/S1084-2756(03)00025-3 15001141

[4] Wolf L Gfroerer S Fiegel H Rolle U. Complications of newborn enterostomies. World J Clin Cases. (2018) 6:1101–10. 10.12998/wjcc.v6.i16.1101 30613668 PMC6306644

[5] Bower TR Pringle KC Soper RT. Sodium deficit causing decreased weight gain and metabolic acidosis in infants with ileostomy. J Pediatr Surg. (1988) 23:567–72. 10.1016/S0022-3468(88)80370-1 2843619

[6] Sacher P Hirsig J Gresser J Spitz L. The importance of oral sodium replacement in ileostomy patients. Progr Pediatr Surg. (1989) 24:226–31. 10.1007/978-3-642-74493-8_25 2513609

[7] O'Neil M Teitelbaum DH Harris MB. Total body sodium depletion and poor weight gain in children and young adults with an ileostomy: a case series. Nutr Clin Pract. (2014) 29:397–401. 10.1177/0884533614528543 24699397

[8] Homan GJ. Failure to thrive: a practical guide. Am Fam Physician. (2016) 94:295–9. 27548594

[9] Laughlin JJ Brady MS Eigen H. Changing feeding trends as a cause of electrolyte depletion in infants with cystic fibrosis. Pediatrics. (1981) 68:203–7. 7267227

[10] Macpherson AJ de Aguero MG Ganal-Vonarburg SC. How nutrition and the maternal microbiota shape the neonatal immune system. Nat Rev Immunol. (2017) 17:508–17. 10.1038/nri.2017.58 28604736

[11] Braniste V Al-Asmakh M Kowal C Anuar F Abbaspour A Toth M. The gut microbiota influences blood-brain barrier permeability in mice. Sci Transl Med. (2014) 6:263ra158. 10.1126/scitranslmed.3009759 25411471 PMC4396848

[12] Marchesi JR The Human Microbiota and Microbiome. Oxfordshire: CABI (2014).

[13] Wall R Hussey SG Ryan CA O'Neill M Fitzgerald G Stanton C. Presence of two Lactobacillus and Bifidobacterium probiotic strains in the neonatal ileum. ISME J. (2008) 2:83–91. 10.1038/ismej.2007.69 18059489

[14] Barrett E Guinane CM Ryan CA Dempsey EM Murphy BP O'Toole PW. Microbiota diversity and stability of the preterm neonatal ileum and colon of two infants. Microbiologyopen. (2013) 2:215–25. 10.1002/mbo3.64 23349073 PMC3633347

[15] Turnbaugh PJ Ley RE Hamady M Fraser-Liggett CM Knight R Gordon JI. The human microbiome project. Nature. (2007) 449:804–10. 10.1038/nature06244 17943116 PMC3709439

[16] Rutayisire E Huang K Liu Y Tao F. The mode of delivery affects the diversity and colonization pattern of the gut microbiota during the first year of infants' life: a systematic review. BMC Gastroenterol. (2016) 16:86. 10.1186/s12876-016-0498-0 27475754 PMC4967522

[17] Robertson RC Manges AR Finlay BB Prendergast AJ. The human microbiome and child growth - first 1000 days and beyond. Trends Microbiol. (2019) 27:131–47. 10.1016/j.tim.2018.09.008 30529020

[18] Backhed F Roswall J Peng Y Feng Q Jia H Kovatcheva-Datchary P Dynamics and stabilization of the human gut microbiome during the first year of life. Cell Host Microbe. (2015) 17:852 10.1016/j.chom.2015.05.012 26308884

[19] Koenig JE Spor A Scalfone N Fricker AD Stombaugh J Knight R. Succession of microbial consortia in the developing infant gut microbiome. Proc Natl Acad Sci USA. (2011) 108 (Suppl. 1):4578–85. 10.1073/pnas.1000081107 20668239 PMC3063592

[20] Pannaraj PS Li F Cerini C Bender JM Yang S Rollie A. Association between breast milk bacterial communities and establishment and development of the infant gut microbiome. JAMA Pediatr. (2017) 171:647–54. 10.1001/jamapediatrics.2017.0378 28492938 PMC5710346

[21] Zoetendal EG Raes J van den Bogert B Arumugam M Booijink CC Troost FJ. The human small intestinal microbiota is driven by rapid uptake and conversion of simple carbohydrates. ISME J. (2012) 6:1415–26. 10.1038/ismej.2011.212 22258098 PMC3379644

[22] Sonnenburg JL Backhed F. Diet-microbiota interactions as moderators of human metabolism. Nature. (2016) 535:56–64. 10.1038/nature18846 27383980 PMC5991619

[23] Osuka A Shimizu K Ogura H Tasaki O Hamasaki T Asahara T. Prognostic impact of fecal pH in critically ill patients. Crit Care. (2012) 16:R119. 10.1186/cc11413 22776285 PMC3580696

[24] Henrick BM Hutton AA Palumbo MC Casaburi G Mitchell RD Underwood MA. Elevated fecal pH indicates a profound change in the breastfed infant gut microbiome due to reduction of bifidobacterium over the past century. mSphere. (2018) 3:e00041–18. 10.1128/mSphere.00041-18 29564397 PMC5853487

[25] Hartman AL Lough DM Barupal DK Fiehn O Fishbein T Zasloff M. Human gut microbiome adopts an alternative state following small bowel transplantation. Proc Natl Acad Sci USA. (2009) 106:17187–92. 10.1073/pnas.0904847106 19805153 PMC2746123

[26] Caspary WF. Physiology and pathophysiology of intestinal absorption. Am J Clin Nutr. (1992) 55:299S−308S. 10.1093/ajcn/55.1.299s 1728844

[27] Duro D Kamin D Duggan C. Overview of pediatric short bowel syndrome. J Pediatr Gastroenterol Nutr. (2008) 47 (Suppl. 1):S33–6. 10.1097/MPG.0b013e3181819007 18667916

[28] Jeppesen PB Spectrum of short bowel syndrome in adults: intestinal insufficiency to intestinal failure. JPEN J Parenter Enteral Nutr. (2014) 38:8s−13s. 10.1177/0148607114520994 24486858

[29] Woolf GM Miller C Kurian R Jeejeebhoy KN. Nutritional absorption in short bowel syndrome. Evaluation of fluid, calorie, and divalent cation requirements. Dig Dis Sci. (1987) 32:8–15. 10.1007/BF01296681 3792183

[30] Booijink CC El-Aidy S Rajilic-Stojanovic M Heilig HG Troost FJ Smidt H. High temporal and inter-individual variation detected in the human ileal microbiota. Environ Microbiol. (2010) 12:3213–27. 10.1111/j.1462-2920.2010.02294.x 20626454

[31] Chang JY Shin SM Chun J Lee JH Seo JK. Pyrosequencing-based molecular monitoring of the intestinal bacterial colonization in preterm infants. J Pediatr Gastroenterol Nutr. (2011) 53:512–9. 10.1097/MPG.0b013e318227e518 21734604

[32] Low JSY Soh SE Lee YK Kwek KYC Holbrook JD Van der Beek EM. Ratio of Klebsiella/Bifidobacterium in early life correlates with later development of paediatric allergy. Benef Microbes. (2017) 8:681–95. 10.3920/BM2017.0020 29022383 PMC5724753

[33] Barreiros Mota I Marques C Faria A Neto MT Cordeiro-Ferreira G Virella D. Colonisation of the proximal intestinal remnant in newborn infants with enterostomy: a longitudinal study protocol. BMJ Open. (2019) 9:e028916. 10.1136/bmjopen-2019-028916 31767579 PMC6886948

[34] Fenton TR Kim JH. A systematic review and meta-analysis to revise the Fenton growth chart for preterm infants. BMC Pediatr. (2013) 13:59. 10.1186/1471-2431-13-59 23601190 PMC3637477

[35] Caporaso JG Lauber CL Walters WA Berg-Lyons D Huntley J Fierer N. Ultra-high-throughput microbial community analysis on the Illumina HiSeq and MiSeq platforms. ISME J. (2012) 6:1621–4. 10.1038/ismej.2012.8 22402401 PMC3400413

[36] Callahan BJ McMurdie PJ Rosen MJ Han AW Johnson AJ Holmes SP. DADA2: high-resolution sample inference from Illumina amplicon data. Nat Methods. (2016) 13:581–3. 10.1038/nmeth.3869 27214047 PMC4927377

[37] Zakrzewski M Proietti C Ellis JJ Hasan S Brion MJ Berger B. Calypso: a user-friendly web-server for mining and visualizing microbiome-environment interactions. Bioinformatics. (2017) 33:782–3. 10.1093/bioinformatics/btw725 28025202 PMC5408814

