# Supplementary figures and images for: The Impact of Oral Sodium Chloride Supplementation on Thrive and the Intestinal Microbiome in Neonates With Small Bowel Ostomies: A Prospective Cohort Study
Source: Front Immunol. 2020 Jul 10;11:1421. doi: 10.3389/fimmu.2020.01421 (PMC7365880; doi:10.3389/fimmu.2020.01421)

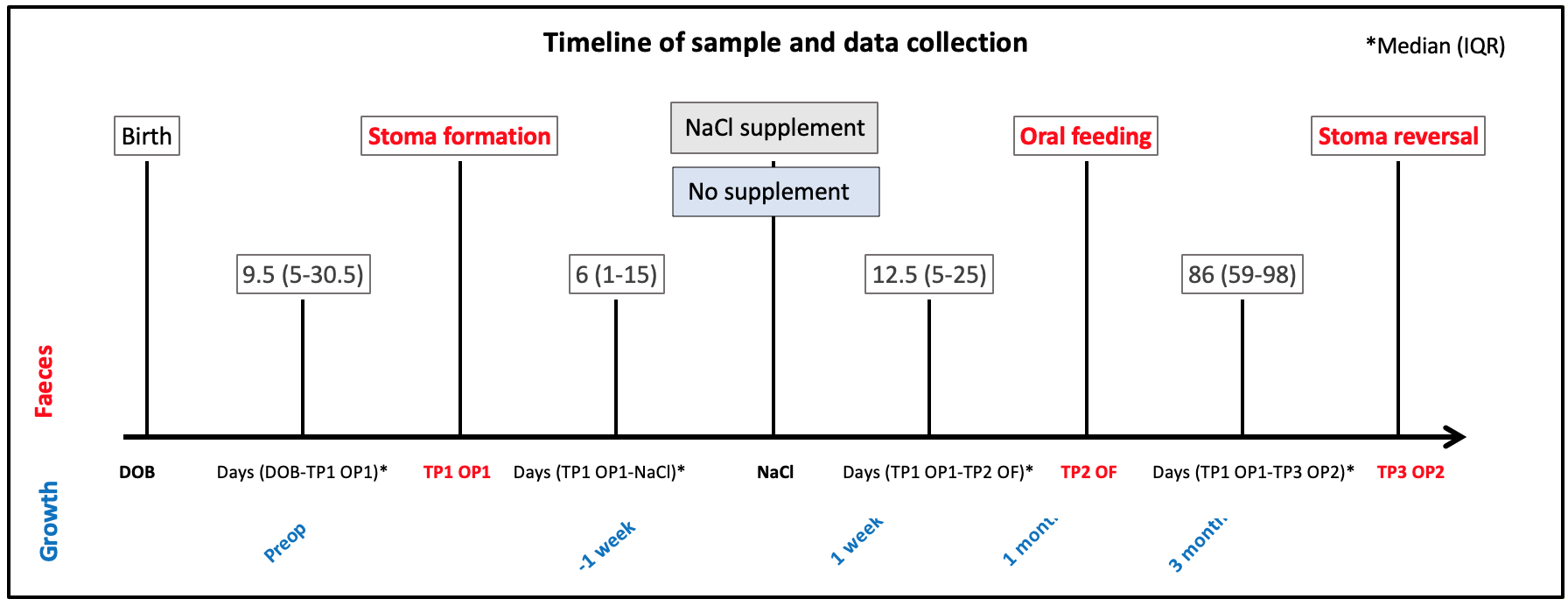

Supplement: Supplement 1 — Timeline of sample and data collection. Red: Fecal samples (resorption, microbiota) were withdrawn on three occasions: after stoma formation (TP1 OP 1), after initiation of oral feeding (TP2 OF), and before stoma reversal (TP3 OP2). Blue: Growth related data (sodium, weight) were documented on five occasions: before stoma formation (preop), 1 week before intervention* (−1 week), and on three occasions after intervention* (1 week, 1 month, 3 months). *intervention = NaCl vs. none. [file Image_1.TIFF]
