# Supplementary material for: The Impact of Oral Sodium Chloride Supplementation on Thrive and the Intestinal Microbiome in Neonates With Small Bowel Ostomies: A Prospective Cohort Study
Source: Front Immunol. 2020 Jul 10;11:1421. doi: 10.3389/fimmu.2020.01421 (PMC7365880; doi:10.3389/fimmu.2020.01421)
Supplement: Supplementary Figure 1 — Number of sequence reads per sample. Brief overview of sequencing reads obtained for samples analyzed in this study. [file Data_Sheet_1.PDF]

# Supplementary Figure 1

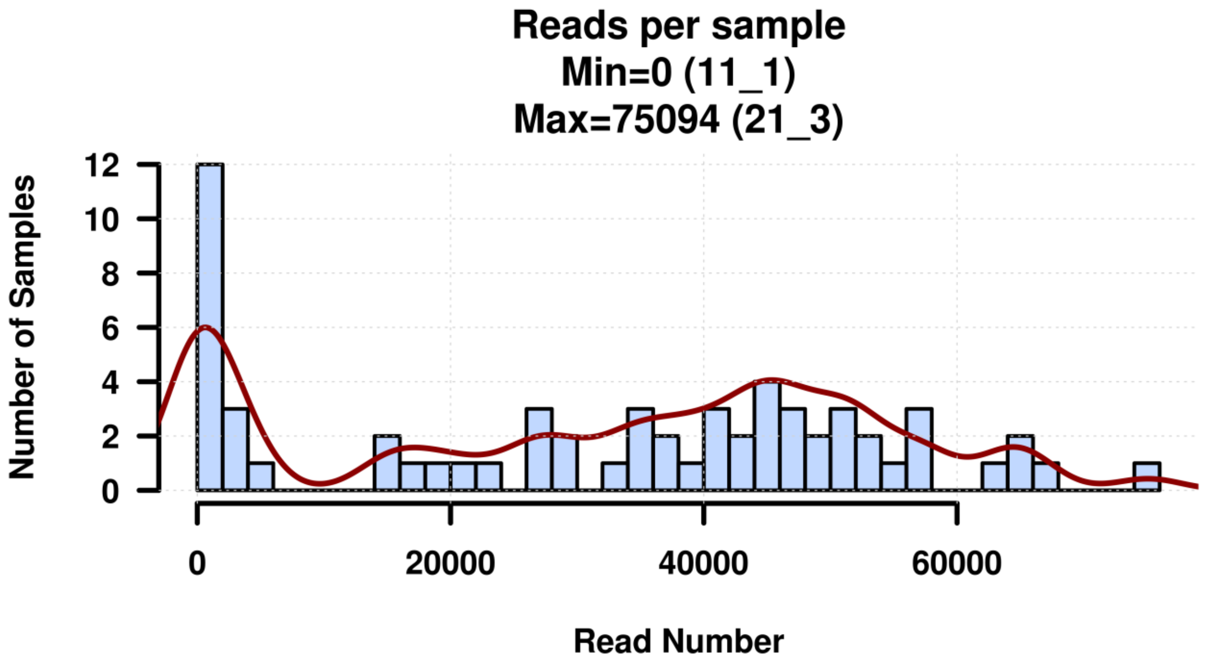


**Suppl. Figure 1: Number of sequence reads per sample.**
Brief overview of sequencing reads obtained for samples analyzed in this study.

# Supplementary Figure 2

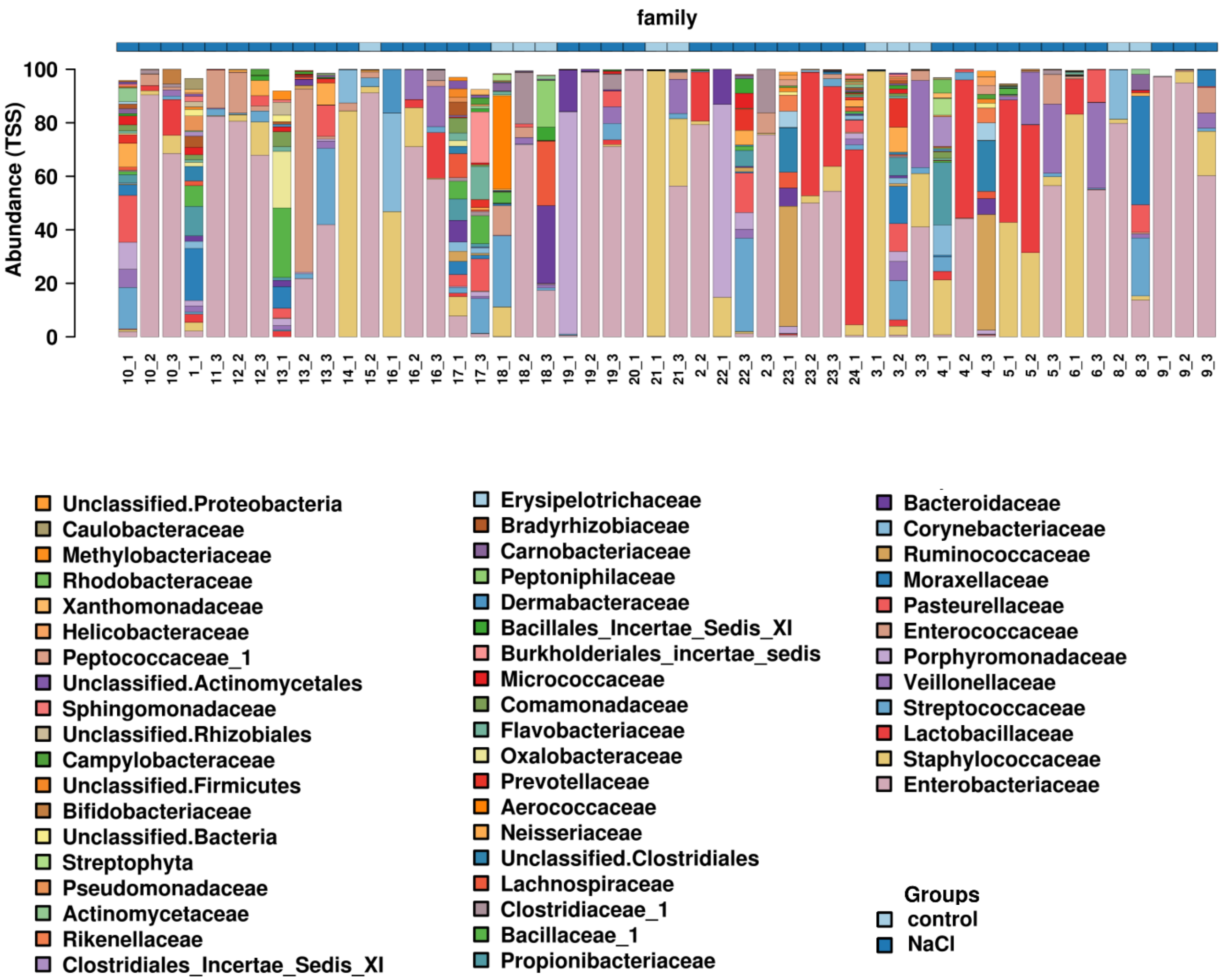


**Suppl. Figure 2: Quantitative visualization of community composition at family level**
Microbial community composition including the 100 most abundant bacterial families is shown for individual samples.

# Supplementary Figure 3

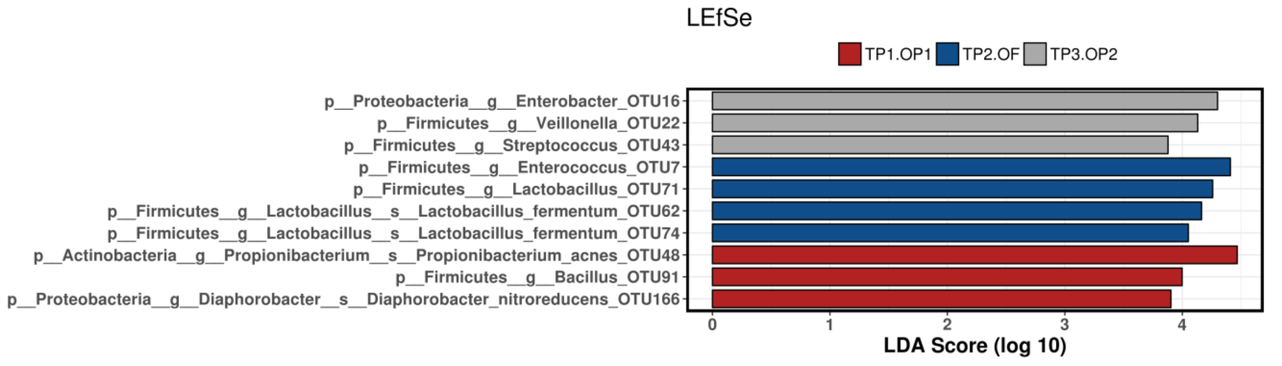


**Suppl. Figure 3: Feature selection for point of times**
Bacterial OTUs associated with different point of times were identified by using the linear discriminant analysis (LDA) effect size method (LEfSe) implemented in Calypso Web version.
